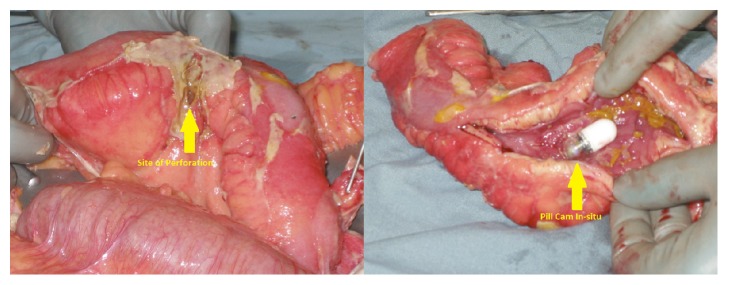# Small Bowel Obstruction with Perforation Secondary to PillCam

**DOI:** 10.1155/2018/9081742

**Published:** 2018-04-08

**Authors:** Toan Pham, Ashley Miller, Domenic La Paglia, Alvin Cham

**Affiliations:** ^1^Department of Surgery, Western Hospital, Melbourne, VIC, Australia; ^2^Department of Gastroenterology, St Vincent's Hospital, Melbourne, VIC, Australia

## Abstract

We present a case of a 63-year-old female who presented with small bowel obstruction and perforation secondary to a retained PillCam®. She initially presented with iron-deficiency anaemia (haemoglobin 44 g/L, ferritin 190 mcg/L). After unremarkable upper and lower gastrointestinal endoscopies and biopsies, she was referred for wireless capsule endoscopy (WCE). Four days afterwards she re-presented with small bowel obstruction and perforation related to the PillCam, which was confirmed on X-ray, on computed topography (CT), and at laparotomy.

## 1. Introduction

PillCam is a capsule endoscopy product used primarily to identify a site of gastrointestinal bleed in cases where both gastroscopy and colonoscopy have been negative. It is a camera capsule that is ingested and takes multiple digital photographs of the bowel during transit. In the vast majority of patients, the capsule is passed without difficulty. However, in some patients, such as those with extensive prior abdominal surgery, small bowel structuring, and radiation enteritis, the capsule can become stuck and potentially cause obstruction. We present a case of a 63-year-old woman who presented with small bowel obstruction and subsequent perforation secondary to a retained PillCam.

## 2. Case Report

A 63-year-old female presented with iron-deficiency anaemia (haemoglobin 44 g/L, ferritin 190 mcg/L). After unremarkable upper and lower gastrointestinal endoscopies and biopsies, she was referred for wireless capsule endoscopy (WCE).

**Figure 1:**
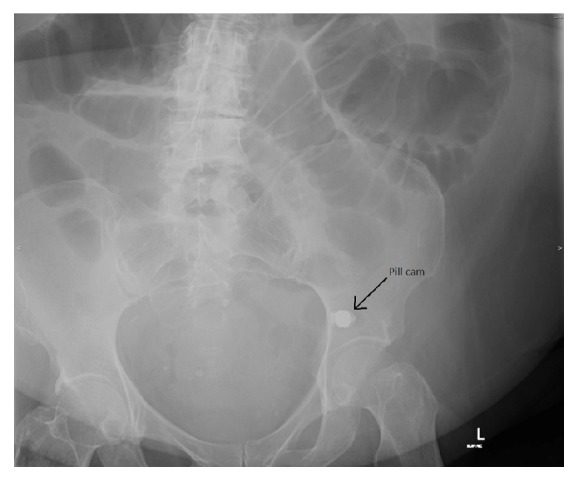


**Figure 2:**
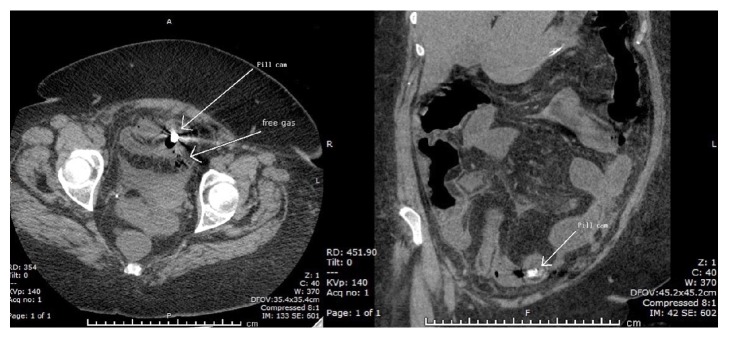


The WCE study demonstrated an area of ulceration and stenosis in the distal jejunum. The capsule failed to pass the pathology within the duration of the capsule's battery life (approximately 14 hours). Four days following ingestion, the patient presented with 48 hours of increasing generalised abdominal pain and nausea, but no vomiting. She had a mild fever of 37.8°C. On examination, the abdomen was distended and tympanic on percussion and generally tender, but there was no guarding. A plain abdominal X-ray (Figure 1) demonstrated multiple dilated loops of small bowel and air-fluid levels, suggestive of a small bowel obstruction. The wireless capsule can be seen in the left lower quadrant of the abdomen. A computed tomography (CT) scan of the abdomen (Figure 2) demonstrated the wireless capsule in the distal ileum, 15 cm proximal to the ileocaecal junction with evidence of perforation at this level. There was diffuse thickening of the terminal ileum distal to site of perforation.

At laparotomy, there was four-quadrant feculent peritonitis. The site of terminal ileum perforation was on the antimesenteric border, 15 cm from the ileocaecal valve, with the wireless capsule in situ (Figure 3). There was a 5 cm segment of markedly thickened ileum distal to the site of perforation. The resected specimen was histologically consistent with Crohn's disease. The patient recovered well and was discharged from hospital five days after her operation.

## 3. Case Discussion

Capsule retention is an uncommon but well recognised complication of WCE occurring in approximately 1.4% of WCE studies. Causes of capsule retention included inflammatory strictures due to Crohn's disease and NSAID enteropathy, as well as malignant lesions [1]. Crohn's disease of the small bowel accounts for 35.3% of all cases of retention [1].

Small bowel obstruction and perforation are rarely seen with capsule retention [2]. However, abdominal adhesions from previous surgeries [2, 3], Crohn's disease stricture [4], and necrotising ileitis [5] have been implicated.

A high index of suspicion as to the possible presence of small bowel stricturing prior to performing WCE is important in reducing the risk capsule retention. Certain conditions may predispose to obstruction and perforation—these include radiation enteritis, large small bowel tumor, extensive abdominal surgery, extensive small bowel or colon diverticulosis, previous GI perforation, and known fistulas. These are manufacturer-indicated contraindications to the use of the PillCam [6].

If small bowel stricturing is suspected, then preliminary investigations including a small bowel contrast series or MRI enterography can be undertaken. In patients with a high index of suspicion for bowel structuring or stenosis, a PillCam Patency Capsule may be given, which is a dissolvable capsule designed to emulate passage of PillCam [7].

## Figures and Tables

**Figure 3:**